# Metabolic Constrains Rule Metastasis Progression

**DOI:** 10.3390/cells9092081

**Published:** 2020-09-11

**Authors:** Niccolo’ Roda, Valentina Gambino, Marco Giorgio

**Affiliations:** 1 Department of Experimental Oncology, European Institute of Oncology-IRCCS, Via Adamello 16, 20139 Milano, Italyvalentina.gambino@ieo.it (V.G.); 2 Department of Biomedical Sciences, University of Padova, Via Ugo Bassi 58/B, 35131 Padova, Italy

**Keywords:** oncometabolism, metastasis, epithelial-mesenchymal transition, cancer therapy

## Abstract

Metastasis formation accounts for the majority of tumor-associated deaths and consists of different steps, each of them being characterized by a distinctive adaptive phenotype of the cancer cells. Metabolic reprogramming represents one of the main adaptive phenotypes exploited by cancer cells during all the main steps of tumor and metastatic progression. In particular, the metabolism of cancer cells evolves profoundly through all the main phases of metastasis formation, namely the metastatic dissemination, the metastatic colonization of distant organs, the metastatic dormancy, and ultimately the outgrowth into macroscopic lesions. However, the metabolic reprogramming of metastasizing cancer cells has only recently become the subject of intense study. From a clinical point of view, the latter steps of the metastatic process are very important, because patients often undergo surgical removal of the primary tumor when cancer cells have already left the primary tumor site, even though distant metastases are not clinically detectable yet. In this scenario, to precisely elucidate if and how metabolic reprogramming drives acquisition of cancer-specific adaptive phenotypes might pave the way to new therapeutic strategies by combining chemotherapy with metabolic drugs for better cancer eradication. In this review we discuss the latest evidence that claim the importance of metabolic adaptation for cancer progression.

## 1. Introduction

The capability of cells to alter their metabolic phenotype according to the surrounding conditions has historically been referred to as metabolic reprogramming [1,2]. Metabolic reprogramming is a key trait of cellular physiology, being involved in several processes such as development [3,4], regeneration [5,6], inflammation [7,8], and cell survival in a stressful microenvironment [9,10]. Cancer cells are constantly exposed to external stress sources, with oxygen and nutrient shortage being a major barrier to tumor survival [11,12]. Therefore, the capability to cope with a harsh microenvironment and thrive represents a major achievement for cancer progression. In this scenario, metabolic reprogramming becomes a relevant cancer hallmark, since it confers a significant advantage over the surrounding environment, allowing cancer cells to adapt and progress [13,14]. Indeed, the plastic and continuous evolution of the metabolic network has been historically related both to cancer cell survival and proliferation [15], metastatic progression [16,17], and even resistance to anti-cancer treatments [18].

Importantly, the understanding of metabolic plasticity in tumor physiology is still puzzling, being dictated by oncogenic signaling, tissue of origin, and even tumor grade [19].

In this scenario, understanding tumor-specific metabolic rewiring and consequent tumor-specific metabolic addiction might provide new actionable targets of clinical relevance [20,21,22].

## 2. The Genetic Roots of Tumor Metabolism Reprogramming

**Figure 1:**
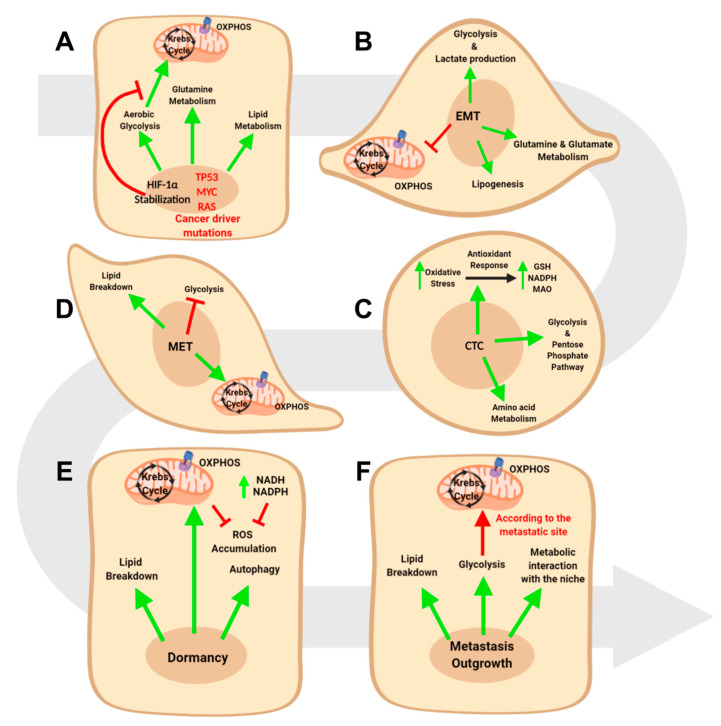
Metabolic reprogramming in cancer progression. (**A**) Driver mutations of cancer leads to metabolic reprogramming in primary tumors. (**B**) epithelial to mesenchymal transition (EMT) progression leads to a further shift towards glycolysis, fatty acid oxidation, and glutamine metabolism. (**C**) circulating tumor cells (CTCs) activate a prominent antioxidant response while, at the same time, maintaining the glycolytic flux. (**D**) MET revert the metabolic reprogramming of EMT, with mitochondrial respiration and lipogenesis upregulation. (**E**) Dormant cells in metastases mainly rely on mitochondrial respiration, autophagy, and fatty acid oxidation. (**F**) The progression towards overt metastases requires a dense interplay with the surrounding niche, with a simultaneous addiction to glycolysis, mitochondrial respiration, and fatty acid oxidation.

The most striking changes in tumor cellular bioenergetics include the elevation of aerobic glycolysis (also termed the Warburg effect), the increase in glutaminolytic flux, the upregulation of amino acids and lipid metabolism, the enhancement of mitochondrial biogenesis, and the induction of a pentose phosphate pathway [23,24] (Figure 1).

These changes are fundamental to sustain tumor proliferation and progression in a genetic context where the major regulators of cell proliferation and physiology are generally mutated [13,14]. For example, glycolytic fueling is associated with activated oncogenes (e.g., *RAS, MYC*), and mutant tumor suppressors (e.g., *TP53*) [25,26,27], whose alterations are pivotal to sustain cell proliferation and attenuate apoptosis.

TP53 mutation is one of the most frequent alterations in human tumors [28], and its role in metabolic reprogramming has been elucidated in several cancers, including hepatocellular carcinoma [29], pancreatic [30], ovarian [31], head and neck [32], and breast cancer [33,34].

TP53 loss can promote glycolysis as a consequence of its role as direct and indirect transcriptional repressor of glucose transporters GLUT1, GLUT4 and GLUT3 [35,36]. In addition, TP53 regulates cell metabolism through the control of TP53-inducible Glycolysis and Apoptosis Regulator (TIGAR) expression: TIGAR is induced by TP53 and displays fructose bisphosphatase enzymatic activity. In particular, TIGAR reduces the amount of intracellular fructose-2,6-bisphosphate, a positive allosteric inductor of glycolysis: therefore, TIGAR mediates a TP53-dependent glycolysis inhibition [37]. In a tumoral TP53-mutated context, the TIGAR-driven brake to glycolysis is removed and cancer cells can upregulate the glycolytic pathway even in the presence of oxygen [38]. TP53 also regulates glucose metabolism through the direct inhibition of glucose-6-phosphate dehydrogenase (G6PD), the first and rate-limiting enzyme in the pentose phosphate pathway [39]. Moreover, the relief of TP53-mediated PTEN induction fosters PI3K-AKT signaling, thus resulting in upregulated glycolysis [40]. Besides glucose metabolism, TP53 was shown to affect glutamine metabolism. In particular, glutaminase 2, which is highly expressed in liver cells, represents a downstream target of TP53. Glutaminase 2 mainly acts by increasing production of glutamate and α ketoglutarate, which in turn leads to enhanced mitochondrial respiration [41]. In hepatocellular carcinoma patients, TP53 mutation is associated with decreased levels of glutaminase 2: functionally, glutaminase 2 suppression is linked to enhanced anchorage-independent survival and increased tumorigenesis in hepatocellular carcinoma [42]. Finally, mutated TP53 promotes fatty acids synthesis through the cooperation with Sterol regulatory elements binding proteins (SREBPs), leading to the upregulation of the mevalonate pathway [43,44].

*MYC* represents another gene frequently overexpressed in many tumors [45], and its role in promoting metabolic reprogramming has been reported in several cancer types, including colorectal [46], pancreatic [47], breast [48], prostate cancer [49], and glioma [50]. In particular, in breast cancer MYC induces the expression of the ADHFE1 oncogene, which upregulates glycolysis, Krebs cycle, and amino acids synthesis [51,52]. Furthermore, *MYC* overexpression has been shown to promote thioredoxin interacting protein (TXNIP) suppression in breast and prostate cancer, thus leading to increased glucose uptake to fuel glycolytic metabolism [48,53]. MYC effects on glycolytic metabolism have been demonstrated also in the setting of glioma, where glycolytic intermediate are used to fuel anabolic purine metabolism [54]. Moreover, two independent works on pancreatic cancer have shown the role of MYC in promoting both glycolysis upregulation [55] and protein anabolism [46]. Lipid-wise, the work from Loda and colleagues showed that *MYC*-overexpressing prostate cancer patients display higher activation of fatty acids turnover with respect to control patients [49], thus suggesting a role for MYC in this branch of cell metabolism. Furthermore, in glioma, the work by Rich and colleagues showed that MYC can upregulate the anabolism of mevalonate, a crucial lipid for cholesterol biosynthesis [50]. Furthermore, MYC was reported to exert a profound effect on glutamine metabolism [56]. Indeed, reverse genetics experiments on glioma cell lines revealed that MYC directly regulate the transcription of high affinity glutamine importers, which are fundamental for glioma cell survival [25]. In neuroblastoma, MYC was shown to upregulate the levels of glutaminase 2, which in turn sustains viability and proliferation of cancer cells. In this scenario, glutaminase 2 depletion strongly impairs the metabolic pathways downstream to glutaminolysis, namely glutamine-dependent anaplerotic reactions and glycolysis [57]. Similarly, studies on lymphoma and prostate cancer demonstrated that MYC promotes the expression of mitochondrial glutaminase through the direct repression of miR-23a and miR-23b, that, in turn, inhibit mitochondrial glutaminase expression. The expression of mitochondrial glutaminase is fundamental for both lymphoma and prostate cancer cell survival and proliferation [58]. Interestingly, MYC was also reported to have a role in promoting glutamine synthesis in a context of glutamine deprivation: in vitro and in vivo experiments on breast cancer cell lines revealed that MYC induces the expression of glutamine synthetase, resulting in increased glutamine synthesis, glutamine-mediated leucine uptake, survival in glutamine-deprived medium, and tumorigenesis [59]. Finally, MYC was also shown to directly regulate proline metabolism in lymphoma and prostate cancer cells through the suppression of proline oxidase and the induction of glutaminase-dependent proline synthesis [60]. Interestingly, MYC-driven proline biosynthesis requires NAD(P)H as cofactor and promotes NAD(P) accumulation: as a function of this recycling, proline biosynthesis interlocks with pathways that generate NAD(P)H, namely glycolysis and pentose phosphate pathway, and ultimately fosters tumor growth [61].

It is worth noting that MYC effects on tumor metabolism are strictly dependent on the cancer’s tissue of origin. Indeed, while MYC-induced murine liver carcinoma displays a significant increase of glucose and glutamine catabolism, MYC-induced lung adenocarcinoma shifts towards glutamine accumulation [19].

Another frequently mutated gene in human cancers is *KRAS*, whose activation leads to deregulated proliferation [62]. This gene has been reported as a major regulator of metabolism reprogramming in several cancers, namely pancreatic [63], colorectal [64], and lung [65]. The main way through which KRAS affects metabolism is via the induction of glycolysis [66] and glutamine metabolism [67], to provide anabolic intermediates to fuel nucleic acids biosynthesis and allow tumor progression. Indeed, similarly to *TP53*, mutated *KRAS* leads to increased expression of the GLUT1, as well as glucose uptake, glycolysis, and lactate production [27]. In addition, in-vitro and in-vivo reverse genetics experiments in pancreatic ductal adenocarinoma (PDAC) revealed that cancer cells rely on a non-canonical pathway of glutamine metabolism, which is strictly regulated by KRAS. Instead of shunting glutamine-derived glutamate into α ketoglutarate in the mitochondria to fuel the tricarboxylic acid cycle, PDAC conveys glutamine-derived aspartate into cytoplasm where it is converted into oxaloacetate by aspartate transaminase. Oxaloacetate is then metabolized to pyruvate, so to provide anabolic intermediates and to increase the NADPH/NADP ratio, which maintains the cellular redox state [67]. Furthermore, KRAS was shown to upregulate the enzyme asparagine synthetase in colorectal cancer via the PI3K-AKT-mTOR pathway, which allows cells to survive and proliferate upon glutamine depletion. Interestingly, *KRAS*-mutated colorectal cancer patients display significantly higher levels of asparagine synthetase enzyme with respect to wild-type *KRAS* patients [68].

Therefore, metabolic reprogramming generally plunges its own roots into a genetically mutated background, where the major drivers of tumorigenesis induce profound changes in metabolic profile.

Eventually, although non mutated in the vast majority of cancer patients [69,70], the Hypoxia Inducible Factor-1α (HIF-1α) represents a critical gene in several solid tumors [71,72]. The main way through which HIF-1α activity is upregulated in cancer is via the post-translational stabilization when the oxygen partial pressure drops below 10 mm Hg [73]. Once stabilized, HIF-1α translocates into the nucleus where it binds to its heterodimeric partner Aryl Hydrocarbon Receptor Nuclear Translocator and then regulates the expression of more than 100 genes [72]. The main effects of HIF-1α on cell physiology involve cell cycle arrest, induction of angiogenesis, and metabolic reprogramming [72,73], and these change have been associated to poor prognosis in several cancer types [74,75,76]. As far as metabolic reprogramming is concerned, HIF-1α was historically associated with a profound upregulation of the glycolytic pathways. First of all, HIF-1α was reported to induce the expression of glucose membrane transporters GLUT1 and GLUT3 [77,78]. Moreover, HIF-1α promotes the overexpression of several enzymes involved in glycolytic steps, namely aldolase A, phosphoglycerate kinase 1, pyruvate kinase M, hexokinase 2, and enolase 1 [79,80,81,82]. In addition, the HIF-1α-mediated glycolytic switch is accompanied by a significant downregulation of mitochondrial respiration [73]. Indeed, HIF-1α was shown to induce the expression of pyruvate dehydrogenase kinase, which in turn inactivates the pyruvate dehydrogenase complex, thus inhibiting the initiation of Krebs cycle [83]. Furthermore, HIF-1α affects mitochondria physiology through the upregulation of the protein BNIP3, whose activity leads to an upregualtion of mitophagic pathway [84]. Eventually, HIF-1α is involved in the direct upregulation of lactate transporters [85] and lactate dehydrogenase A, which converts the glycolytic pyruvate into lactate and restores the intracellular NAD [86].

In parallel, HIF-1α was reported to affect fatty acid metabolism and, in particular, to promote lipogenesis at the expense of fatty acid oxidation. Indeed, besides inhibiting pyruvate dehydrogenase, HIF-1α counters fatty acid oxidation, another major source of acetyl-CoA, through the transcriptional repression of medium- and long-chain acyl-CoA dehydrogenases [87]. On the other hand, HIF-1α was shown to upregulate the expression of SREBP-1, which in turn promotes the expression of fatty acid synthase, thus indirectly promoting a lipogenic shift in hypoxic cells [88]. Besides lipid synthesis, HIF1-α was also reported to increase the lipid levels of cancer cells through the direct upregulation of Fatty Acid Binding Protein 3 (FABP3) and FABP7, both involved in lipid uptake. In addition, uptaken lipids are generally conveyed into lipid droplets during hypoxia and, coherently, HIF-1α was shown to upregulate adipophilin, an essential structural component of lipid droplets [89]. Interestingly, the effects of HIF1-α on lipid metabolism interlock with the effects on amino acid metabolism. Indeed, HIF-1α was shown to induce the expression of SIAH2, which in turn promotes the proteolysis of OGDH2, a critical subunit of the enzyme complex α ketoglutarate dehydrogenase [90]. In this way, α ketoglutarate cannot fuel the Krebs cycle and is shunted towards lipid biosynthesis through the activity of isocitrate dehydrogenase 1: interestingly, the α ketoglutarate which is used to fuel lipogenesis is mostly derived from glutamine catabolism in hypoxic conditions [91]. Furthermore, hypoxia was reported to upregulate glutamine and leucine uptake in neuroblastoma cell lines, thus rising the intracellular amino acid availability [92]. Similarly, a recent work on glioblastoma (GBM) revealed that HIF-1α fosters branched-chain amino acid (BCAA) metabolism by upregulating the expression of both BCAA transporter LAT1 and BCAA metabolic enzyme BCAT1 [93]. Eventually, studies on hepatocellular and renal carcinoma cells revealed that HIF-1α induces glutamate release by increasing the expression of glutamate transporters SLC1A1 and SLC1A3. At the same time, HIF-1α also upregulates the expression of glutamate receptors, thus fostering a glutamate signal in cancer cells and ultimately leading to tumor outgrowth [94].

## 3. Metabolic Intra-Tumor Heterogeneity

Tumor limitless proliferative potential arises in a genetically mutated background, which allows cancer cells to overcome proliferation barriers in response to intrinsic and extrinsic perturbation [95,96]. However, although being generated by a single cell, tumors become complex ecosystems composed of extremely different cells. This phenomenon—generally referred to as intra-tumor heterogeneity—has been extensively reviewed and involves both a genetic and epigenetic counterpart [97]. Indeed, overt tumors are characterized by the presence of cells that differ both under the mutational profile and under the transcriptional profile [98].

**Figure 2:**
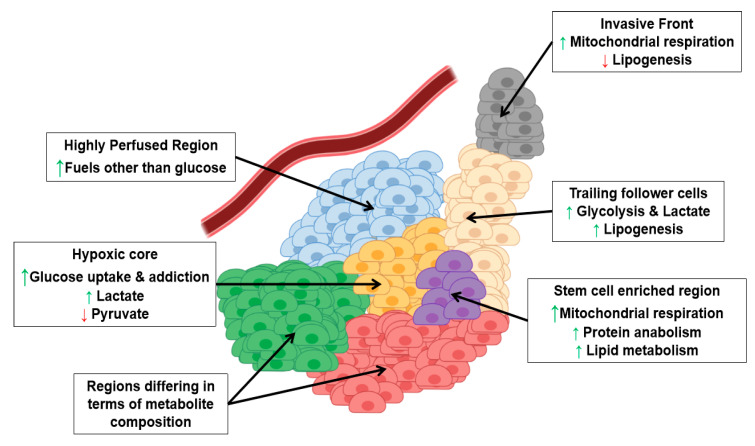
Metabolic Heterogeneity of cancer lesions. Tumoral cells experience different metabolic profile according to spatial and functional needs (the green arrow up indicates increase, the red arrow down indicates decrease).

Intriguingly, intra-tumor heterogeneity is not limited to these two aspects, being also tightly bound to the metabolic profile of cancer cells (Figure 2). Metabolic heterogeneity is crucial for several aspects of human physiology since it allows closely related cells to take on distinct tasks [99,100,101].

Similarly, multiple cancers have been shown to be metabolically heterogeneous, with a distinct part of tumoral tissue activating distinct metabolic pathways. In this way, cancer cells dynamically adapt to and cope with the surrounding microenvironment where nutrient and oxygen shortage imposes the activation of and the addiction to specific metabolic pathways [102]. Then, tumor metabolism is spatially dictated by the interaction with the surrounding microenvironment, where cancer associated fibroblasts, blood vessels, and immune cells are heterogeneously distributed [103,104].

Single-cell gene expression profiling of head and neck squamous cell carcinoma (HNSCC) and melanoma revealed that cells from the same tumor may significantly differ in the expression of genes involved both in oxidative phosphorylation (OXPHOS) and glycolysis [105]. Moreover, HNSCC’s metabolic heterogeneity can be fostered by stress conditions: in particular, chemotherapy administration was shown to increase spatial metabolic heterogeneity in HNSCC, with the segregation of different tumoral populations characterized by differential NADPH stability [106]. In addition, the in-vivo application of oxygen sensitive dyes in HNSCC tumors revealed that glycolytic fluxes are higher in hypoxic regions with respect to normoxic ones [107]. Similarly, the metabolic analysis of one renal cell carcinoma patient revealed a marked regional distribution of pyruvate and lactate, with lactate increasing and pyruvate decreasing in the presence of hypoxic regions [108]. Moreover, a work on human breast cancer showed that the inner part of the tumor is more oxidized and less capable to uptake glucose than the periphery, despite the presence of islets of cells in the necrotic core that exhibit high glucose uptake [109]. Intriguingly, cells derived from a single breast cancer patient biopsy were shown to display a significantly higher heterogeneity in oxygen consumption rate (and, therefore, in metabolism [110]) when compared to normal cells from the same patient [111]. Spatial metabolic heterogeneity was also reported in lung cancer. First of all, the work by DeBerardinis and colleagues proved that cancer cells within the same lesion can fine-tune their metabolism according to the external nutrient supply. In particular, cells that experience low perfusion display higher glucose addiction to fuel their metabolic machinery, whereas highly perfused cells can switch onto different carbon sources, including amino acids, lipids, ketones, and lactate [112].

In addition, metabolic analysis of renal cell carcinomas indeed revealed the presence of two distinct metabolic clusters within a single tumor, one characterized by glycolytic and pentose phosphate intermediates and another characterized by cystine and 2-oxobutyric acid and depleted for glycolytic intermediates [113]. In the same way, spatial analysis of esophageal cancer showed that metabolite profile can be extremely variable within the cancerous tissue: in particular, metabolites such as amino acids, fatty acids, and nucleosides were found to be unevenly distributed in the the tumoral milieu [114]. Heterogenous metabolite distribution within single tumors was also confirmed in colorectal [115], gastric [116], prostate [117], and papillary thyroid cancers [118].

Metabolic heterogeneity has been shown as a peculiar trait also of hematologic malignancies, as reported by two studies on lymphoma by Martinez-Outschoorn and colleagues. Here, the metabolism of tumor cells has been shown to differ from the stromal one (i.e., tumor associated macrophages, fibroblasts, neutrophils, and many others). In particular, lymphoma cells are characterized by oxidative metabolism, while stromal cells display a tumor-induced glycolytic metabolism. In addition, stromal cells actively export lactate via MCT-4 transporter and the molecule is rapidly uptaken by tumoral cells via MCT-1: in this way, lactate fuels the OXPHOS of tumoral cells [119,120].

In addition to spatial metabolic heterogeneity, the work by Lisanti and colleagues showed a functional metabolic heterogeneity, with breast cancer stem cells upregulating mitochondrial proteins, glycolysis, and protein anabolism enzymes with respect to the rest of the tumor [121]. Functional metabolic heterogeneity of cancer stem cells has also been reported in brain tumors. In particular, GBM slow-cycling cells were proven to display higher activation of mitochondrial OXPHOS and lipid metabolism with respect to fast cycling cells [122]. In addition, two independent works have shown that GBM stem cells (GSCs) may exist in two different states: when subjected to hypoxia, GSCs activate glycolysis, whereas they turn to OXPHOS when oxygen becomes available [123,124].

Cancer cells may display extreme metabolic dissimilarity even in a narrow space interval. Indeed, a study conducted on the invasive front of lung cancer showed that the leading cells of the migratory front specifically rely on mitochondrial respiration, whereas the trailing follower cells activate glycolysis [125]. This suggests an extremely precise fine-tuning of metabolic properties of lung cancer cells during stromal invasion.

Moreover, progression from early to advanced lung cancer is usually associated with a change in the genetic profile, with heterozygous KRAS mutation becoming homozygous. In this scenario, two distinct populations coexist and have been shown to differ under the metabolic profile, with homozygous cells displaying increased glycolysis, glucose uptake, and lactate secretion with respect to heterozygous ones [126].

This dynamic evolution of metabolic profile is also shared by melanoma, where metastasis spreading has been linked to metabolic features that distinguish cellular populations. Indeed, the progression towards an invasive phenotype in melanoma has been linked to the downregulation of the microphthalmia-associated transcription factor (MITF) oncogene, which in turn leads to reduced lipogenesis [127]. Therefore, invasive non-lipogenic melanoma cells coexist with lipogenic proliferative cells within the same lesion. In addition, melanoma metastasis has been also linked to lactate metabolism, with the lactate transporter MCT1 being upregulated in metastasizing melanoma cells [128]. Intriguingly, lactate metabolism was also identified as a key player in oral squamous cell carcinoma, with lactate uptaking cells being more proliferative than the surrounding lactate exporting cells [129].

Finally, a recent work on PDAC demonstrated how epigenetic mechanism accounts for the metabolic plasticity and heterogeneity of tumoral cells. In particular, the authors show that a spontaneous dysregulation of the SWI/SNF remodeling complex causes a de-differentiation of a subpopulation of pancreatic tumor cells into a more aggressive and mesenchymal one. The resulting mesenchymal tumoral cells are characterized by the depletion of the chromatin regulator SMARCB1 and by the consequent activation of MYC, eventually leading to increased protein anabolism and biomass synthesis [47].

## 4. Metabolic Reprogramming in Epithelial to Mesenchymal Transition

Metabolic reprogramming represents an efficient response of tumoral cells to a hostile microenvironment. However, the persistence of harsh conditions has been shown to be detrimental for tumor progression, leading to proliferation arrest and, eventually, cell death. In particular, a tumor microenvironment is characterized by deregulated cellular proliferation, which in turn leads to nutrient deprivation and oxygen consumption [130]. Glucose shortage has been shown to induce cell cycle arrest [131] and apoptotic cell death [132,133] in in-vitro cancer models. In the same way, glutamine deficiency exerts the same effect on tumoral cells: in-vitro studies conducted on neuroblastoma and breast cancer cell lines have shown that glutamine deprivation leads to apoptosis [134] and proliferation arrest [135], respectively. On top of that, severe hypoxia conditions have been shown to promote cancer cell death in in-vitro cancer cell lines [136].

In addition to the effects on tumoral cell death and proliferation, the harsh microenvironment plays a role in promoting the epithelial to mesenchymal transition (EMT). In particular, glucose deficiency promotes invasive traits [137] and EMT induction [138]. On the other hand, glutamine shortage increases the expression of the asparagine synthetase enzyme [139], which in turn promotes EMT and metastasis [140]. Ultimately, in-vitro hypoxic conditioning boosts EMT through the unfolded protein response (UPR)-dependent release of Transforming Growth Factor β (TGFβ) [141].

Therefore, tumor cells experience a stressful microenvironment that induces cell cycle alteration and, concomitantly, fosters invasive traits. In this scenario, EMT represents a way through which cancer cells may avoid the detrimental effects of stress: several works have pointed out that the acquirement of stemness traits in EMT counters the induction of cell death upon harsh conditioning [142,143,144].

### 4.1. Glucose Metabolism and EMT

Interestingly, the EMT process involves a profound rearrangement of cellular metabolism across different tumor types (Figure 1A), with a complex network of pathways being perturbed by the major regulators of this process, such as Twist1, Snai1, and Zeb1 [17,145]. One of the most reported events in EMT is the increase of glucose uptake and of glycolytic pathway [146], which derives from the activity of several EMT regulators. In particular, pancreatic ductal carcinoma cells exposed to the EMT-inducers Tumor Necrosis Factor α (TNFα) and TGFβ upregulate the expression of glucose transporters GLUT1 and GLUT 3 and the expression of several glycolytic enzymes (i.e., HK2, PKM2, LDH-A, and LDH-B) [147]. In parallel, experiments on breast cancer cell lines showed that Twist1 overexpression induces similar effects, thus increasing glucose uptake and glycolytic fueling [148]. In-vitro experiments in breast [149] and lung cancer cell lines [150] have also shown that glucose transporters represent Zeb1 transcriptional targets. On top of that, by a reverse genetics approach, it has been shown that the EMT inducer Fyn-related kinase (FRK) promotes glucose uptake and glycolytic metabolism in lung cancer models [151]. Coherently, the overexpression of the EMT effector Snai1 promotes glycolysis through the inhibition of the fructose-1,6-biphosphatase enzyme [152] and through the upregulation of glucose transporters [153].

These works suggest that EMT requires increased glucose supply to guarantee cell survival. Interestingly, the work of Kim and colleagues has shown a completely different situation, with Snai1 repressing the rate-limiting glycolytic enzyme phosphofructokinase-1 to shunt glucose towards the pentose phosphate pathway [154].

Besides glycolysis upregulation, increased lactate production has been reported as a common marker of EMT metabolic reprogramming [17]. Indeed, works on pancreatic [147,153], breast [148,149,150,151,152], and lung cancers [151], as well as hepatocellular carcinoma [155] have indeed shown an increase in lactate production in highly glycolytic cancer cells experiencing EMT.

As a consequence of glycolytic promotion, a reduced activity of mitochondrial function in EMT-experiencing cancer cells [145] has been reported. Indeed, the analysis of a panel of twenty different solid tumors revealed a significant downregulation of Krebs cycle and OXPHOS genes in the presence of EMT signature expression [156]. In addition to this, an in-vitro study on lung cancer cell lines showed that TGFβ-mediated EMT increases mitochondrial reactive oxygen species (ROS) production and induces mitochondrial membrane potential drop [157]. Moreover, in-vitro works with breast cancer models showed that basal cell lines display significantly reduced mitochondrial function when compared with luminal cell lines [158]. On the other hand, in-vivo models of breast cancer have provided significant evidence that downregulation of the mitochondrial protein TMEM126A leads to increased invasiveness and metastasis [159]. Therefore, these works corroborate the fact that an efficient EMT should rely on glycolytic rather than on oxidative metabolism.

### 4.2. Lipid Metabolism and EMT

In addition to glucose metabolism, EMT has been shown to alter the lipid profile of cancer cells [146]. In particular, during EMT the metabolism of lipids has been reported to shift towards lipogenesis, to the detriment of fatty acid oxidation [145]. Indeed, Liang and colleagues showed in a cohort of salivary adenoid cystic carcinoma patients that high levels of PRRX1, a potent EMT inducer, positively correlate with free fatty acids accumulation [160]. Furthermore, increased levels of sphingosine 1-phosphate positively correlate with enhanced EMT in colorectal [161,162] and bladder cancer [163]. To further corroborate a role for lipogenesis in fostering an efficient EMT, a work by Xing and colleagues showed that lipid catabolism severely impairs EMT in clear cell renal carcinoma [164].

A study conducted on colorectal cancer provided a causal role for the lipogenic pathway operated by the acyl-CoA synthetase/Stearoyl-CoA desaturase network in promoting EMT switch and invasiveness [165]. On top of that, a work on lung cancer cell lines demonstrated a pro-EMT role for the ATP citrate lyase enzyme, which converts citrate to acteyl-CoA, thus generating the building blocks for lipogenesis [166]. Coherently, augmented fatty acid synthase (FASN) activity and cholesterol biosynthesis enhance the expression of EMT positive regulators and induce metastases in ovarian and breast cancer models [167,168]. Then, another study on lung cancer cell lines showed that FASN can promote TGFβ signaling, thus reinforcing the EMT process [169]. Conversely, other works have pointed out that FASN activity has to be reduced in order to efficiently perform EMT in breast [170] and lung cancer cell lines [171]: a possible explanation may be that FASN activity is required to trigger EMT, while it has to be inhibited in order to complete the transition [145].

### 4.3. Amino Acid Metabolism and EMT

Together with carbohydrates and lipid metabolism, amino acid and, in particular, glutamine metabolism plays a key role in EMT [17]. Indeed, high intracellular glutamine levels were shown to be necessary to promote EMT in several in-vitro models [172]. In particular, efficient glutamine uptake via SLC1A5 transporter is fundamental for prostate cancer proliferation and metastasis via the upregulation of E2F-dependent cell cycle genes [173]. Glutamine can promote EMT through the enzyme glutaminase, that converts glutamine to glutamate. Indeed, works on lung [174] and ovarian cancer [175] showed that invasive phenotype strongly relies on the expression of glutaminase. In this regard, a study by Kang and colleagues showed that EMT phenotype in breast, ovarian, and colorectal cancers depends both on glutamine levels and glutaminase activity [176]. In parallel, a recent study on colorectal cancer patients demonstrated that the enzyme glutamate dehydrogenase is fundamental to mediate metastasis via EMT [177]. Conversely, a work by Mani and colleagues suggested that breast cancer needs to become glutamine independent to efficiently perform EMT [178]. Also in this case, a possible explanation can be a different role for glutamine in EMT initiation and completion.

Therefore, cancer cells should accomplish stringent metabolic requirements to successfully perform the EMT. In particular, the glycolytic switch that is usually associated with tumor development is further exacerbated in EMT, with a concomitant reduction in mitochondrial activity. Similarly, tumors progressing through EMT foster the glutamine dependency that already characterizes the cancer growth. Ultimately, lipid metabolism is funneled towards lipogenesis, with fatty acid levels being essential for EMT to progress.

## 5. The Metabolic Reprogramming in Circulating Tumor Cells and Mesenchymal to Epithelial Transition

### 5.1. Metabolic Requirements of Circulating Tumor Cells

Metastasis spreading is a multi-step process which also requires tumoral cells to survive in the bloodstream and then colonize specific organ niches. Previous works described the metabolic requirements that circulating tumor cells (CTCs) fulfill in order to survive in the circulation (Figure 1C).

First of all, matrix detachment and nutrient paucity induce an increased oxidative stress in tumor cells [179,180], and, therefore, CTCs need to activate an antioxidant response to survive. In line with this, two in vitro studies from DeBerardinis and colleagues showed that antioxidant response improved the survival in melanoma and lung cancer models upon matrix detachment. Melanoma cells were proven to reprogram their metabolism and activate the NADPH-generating folate pathway: NADPH is shunted to regenerate glutathione supply, which is fundamental to withstand oxidative stress [181]. Similarly, lung cancer cells were shown to repress the oxidative metabolism of glucose and glutamine and to promote the reductive formation of citrate from glutamine, which increases the production of NAPDH and the concomitant buffering of ROS [182]. Also, an in-vitro work conducted with colon cancer cells investigated the differences in the gene expression profile between the metastasis-competent CTC-MCC-41 cell line and the primary-tumor derived HT-29 cell line [183]. The two models were revealed to be extremely different under the metabolic profile, with the CTC-spreading cell line upregulating fatty acid oxidation and enzymes traditionally involved in ROS scavenging (i.e., monoamine oxydase (MAO) [184], paraoxonase [185], and glutathione S-transferase [186]).

In addition to antioxidant response, several works showed that CTC largely rely on glucose metabolism. In particular, the glycolytic enzyme PGK1 and the pentose phosphate pathway enzyme G6PD were shown to be a reliable marker to isolate aggressive CTC subpopulations in a cohort of breast cancer patients [187]. Coherently, the upregulation of the same gene represents an efficient strategy to identify metastatic prostate cancer patients [188]. The high glycolytic metabolism has also been proven to result into a high lactate production, which was indeed used in devices aimed at CTC isolation [189,190]. Ultimately, a work from Aceto and colleagues conducted in CTCs derived from breast cancer patients pointed out a significant hyperactivation of the ATP metabolic process and amino acid metabolism [191].

### 5.2. Metabolic Requirements of the Mesenchymal to Epithelial Transition

Upon dissemination into the bloodstream, cancer cells revert the EMT to colonize organs and spread metastases, a process known as mesenchymal to epithelial transition (MET) [192]. Similar to EMT, MET requires a profound metabolic reprogramming (Figure 1D).

First of all, one of the major metabolic changes of EMT, increased glycolysis addiction, has been shown to decrease in the MET process. In particular, works on breast [193], ovarian [194], and lung cancer [195] showed that glycolysis inhibition is markedly associated with EMT repression, thus resulting in MET promotion. A logic counterpart to glycolysis inhibition is represented by oxidative metabolism, which is indeed strongly upregulated in MET accomplishment. The work by Kralli and colleagues showed that in vitro upregulation of the Estrogen-Related Receptor γ in breast cancer cells results in an increased oxidative capacity and MET promotion, ultimately affecting tumor fitness [196]. Similarly, experiments on rat adenocarcinoma showed that a hyperoxic state induces glycolysis repression and oxidative metabolism promotion, which in turn are associated with MET [197]. These works suggest that the reversion of EMT glycolytic metabolism back to OXPHOS is a key process to promote MET.

In addition to glucose, lipid metabolism has been shown to play a role in MET promotion. Indeed, in-vitro [198] and in-vivo [199] studies on breast cancer revealed that increased lypolysis is a key trait of cells that revert the EMT phenotype. These works further corroborate previous experiments, where lipid oxidation was shown to be crucial in EMT suppression [200,201].

## 6. The Metabolic Evolution of Metastasis Outgrowth

The colonization of distant organs and the formation of overt metastases both require a dynamic interaction between the tumor cells and the surrounding microenvironment. The specific organs colonized by a tumor type collectively define the tropism for that tumor, with lungs, liver, brain, bones, and lymph nodes being generally the most colonized organs across cancer types [202].

### 6.1. Metabolic Features of In Situ and Metastatic Dormant Cancer Cells

Metastasis spreading requires a dense interplay between tumoral and stromal cells, based both on metabolic and non-metabolic features. However, the progression from CTCs to overt metastasis represents a generally long-lasting and complex phenomenon. This progression passes, in most cases, through a dormancy phase [203] where cancer cells survive in a non-proliferative state in the organ niche, again relying on a dramatic rearrangement of metabolism (Figure 1E). Interestingly, cancer dormancy metabolism is differently regulated during cancer progression. Indeed, two distinct studies on acute myeloid leukemia proved that dormant cells strictly decrease the levels of ROS through the activation of OXPHOS metabolism, at the expense of glycolysis [204,205]. This fact is somehow counterintuitive, since OXPHOS is generally reported to increase the levels of ROS [206]. To better understand this point, it is worth mentioning that cancer cells are generally characterized by high metabolic rate, with extremely elevated ROS production as a consequence. In rapidly growing tumors, ROS-mediated DNA damage provides a selective advantage to cancer cells, since it promotes mutations, DNA instability, and eventually development of chemoresistance [207]. Several in vitro studies, showed that the inhibition of mitochondrial electron transport chain fosters ROS accumulation, leading to apoptotic cell death [208,209,210]. Therefore, in a dormant system, the reduction of ROS can be successfully achieved through the dampening of the metabolic rate and through an efficient OXPHOS. Similarly, dormant glioma stem cells were proven to shunt glycolytic intermediates towards glycerol and phospholipid metabolism via the upregulation of the enzyme GPD1: this metabolic profile is crucial to withstand ROS and ultimately maintain the dormant compartment of glioma cells [211]. ROS withstanding was shown to be critical also in in vitro models of dormant breast cancer, where a generally slower metabolism is associated with glycolytic downregulation and nicotinamide synthesis upregulation: ROS detoxification is achieved here through the increased production of NADH and NADPH [212]. At the same time, pancreatic ductal adenocarcinoma stem cells show metabolic addiction to OXPHOS, fatty acid oxidation and autophagy in order to survive and promote tumor relapse [213]. Furthermore, fatty acid oxidation was demonstrated to be crucial also in in-vitro and in-vivo breast cancer dormancy models [214]. Finally, autophagy represents a key pathway for ovarian cancer dormant cells [215]: interestingly, the autophagic switch leads to glycolysis upregulation in this tumoral setting, thus suggesting a model-dependent metabolic profile in the process of dormancy establishment.

The metabolism of dormant cells was also investigated in metastatic settings, where tumoral cells reprogram their metabolic profile to successfully cope with the new microenvironment. Dormant pancreatic ductal adenocarcinoma metastases to liver activate oxidative phosphorylation in the absence of inflammation, while they shift towards glycolytic and proliferative phenotype when inflammation rises [216]. In an opposite way, in-vitro models of breast cancer bone metastasis showed that dormant cancer cells are generally better maintained in an overall glycolytic microenvironment, while an oxidative microenvironment leads to breast cancer outgrowth [217]. Similar results were also obtained in colorectal cancer models, where prominin-dependent glycolysis upregulation was identified in the highly metastatic dormant cancer cells [218]. These works suggest an extreme diversity in the metabolic program of dormant metastatic cells, which depends both on the tissue of origin and on the metastatic niche. Similarly to in situ ovarian cancer dormant cells [215], two works from Shepherd and colleagues showed that dormant metastatic ovarian cancer cell viability relies on autophagy upregulation via LMP1-AMPK signaling [219,220]. To further corroborate this notion, dormant breast cancer cells that metastasize to the lung were proven to depend on a autophagy to survive [221].

### 6.2. Metabolic Requirements in Metastatic Outgrowth

Eventually, cancer cells evade the dormant state to become overt metastases (Figure 1F). First of all, metastatic cells can revert the dormant phenotype through a molecular dialogue triggered by the metastatic stroma. For example, neutrophil extracellular traps elicit proliferation of lung cancer dormant cells via the proteolytic remodeling of extracellular laminin that activates the YAP signaling [222]. In addition, breast cancer metastasis to lungs were shown to progress via GALNT14-mediated signalling, which overcomes the inhibitory signals of lung bone morphogenetic proteins and promotes the establishment of a favorable microenvironment in the lungs [223]. Ultimately, astrocytes promote the proliferation of melanoma and breast cancer brain metastases through the supply of fatty acids that can be metabolized by cancer cells [224].

Specific metabolic programs are then required to foster the proliferation of the awaken dormant cancer cells. A recent work on breast cancer unveiled the metabolic profile of lung micrometastases: breast cancer cells were shown to upregulate the OXPHOS pathway, with amino and fatty acid metabolism also converging towards mitochondrial respiration [225]. Also, pyruvate metabolism was reported as a key player in fostering breast cancer metastases to lungs. Indeed, pyruvate can fuel aerobic respiration through pyruvate carboxylase and therefore allow ROS withstanding [226]. On top of that, pyruvate uptake also induces the production of oxoglutarate, a key intermediate for collagen biosynthesis and, consequently, for collagen-mediated niche shaping [227]. In addition to pyruvate, fatty acid oxidation was shown to promote breast cancer lung and liver metastases through the activation of Src oncogene: intriguingly, ROS scavenging represents an important step to be achieved in order to have Src activation [228]. Coherently, breast cancer metastases to lymph nodes were shown to rely on the upregulation of YAP-mediated fatty acid oxidation to successfully outgrow [229]. Moreover, also in oral carcinoma and gastric cancer, the upregulation and post-translational modification of the fatty acid receptor CD36 was shown to foster fatty acid oxidation, ultimately promoting lung [230,231] and omental fat pad metastases [232].

Similar to what has been observed in metastatic breast cancer dormant cells, the murine breast cancer cell line 4T1 adopts different behaviors when reaching distinct metastatic sites, with liver metastases showing a glycolytic phenotype, while lung and bones metastases move towards OXPHOS [233]. As for breast cancer metastases, also melanoma-derived metastases were proven to be metabolically heterogeneous, with brain metastases upregulating OXPHOS more than extracranial metastases [234].

Interestingly, in the brain niche, breast cancer metastatic cells establish a functional crosstalk with stromal cells: on one hand, breast cancer suppress the glucose uptake in non-tumoral cells via miR-122 secretion, thus increasing nutrient availability [235]; on the other hand, breast cancer cells acquire brain-like properties, switching onto a GABAergic phenotype that allows to uptake and catabolize GABA [236]. Besides breast cancer, other tumor types were investigated in the metabolic reprogramming process that is involved in overt metastasis establishment. A recent work from Lengyel and colleagues reported an active interaction between ovarian cancer metastases and adipocytes in the omental fat pad. In particular, the elevated fatty acids supply from adipocytes is exploited by metastatic cells that rely on fatty acid oxidation to proliferate [237]. Eventually, a work on colorectal cancer metastases showed that the liver parenchyma upregulates ALDOB-mediated fructose metabolism in tumoral cells. As a consequence, glycolysis and pentose phosphate pathways are fostered specifically in liver metastases, where they promote colorectal cancer metastasis growth [238].

## 7. Metabolism and Cancer Therapy

Resistance to available standard of care therapies represents a major hurdle in cancer eradication [239]. In clinical settings, the persistence of tumor cells that were not eliminated by chemotherapy generally leads to cancer relapse, with tumoral cells remodeling their phenotype upon treatment and becoming even more complex to eradicate [240]. Interestingly, metabolism reprogramming has been shown to be relevant also in the process of therapy resistance, with biochemical pathways being extremely fine-tuned by the chemotherapy bottleneck. Therefore, metabolism relevance in tumor progression has been envisaged as a double-edged sword: indeed, the metabolic addiction of cancer cells can be further exploited to incisively counter tumor progression and metastatic relapse.

Even in non-tumoral settings, doxorubicin and cisplatin were proven to induce a profound rearrangement of cellular metabolism. Indeed, in vitro models of mouse embryonic stem cells showed that cisplatin administration promotes significant changes in nucleotide and amino acid metabolism and in urea cycle [241]. In parallel, rats treated with doxorubicin displayed reduced adipogenesis, with the major regulator PPARγ being severely downregulated by the drug. On top of that, PPARγ inhibition was demonstrated to impede plasma glucose and lipid clearance, thus resulting in hyperglycemia and hyperlipidemia [242].

Similarly, in tumoral settings, chemotherapy was proven as a major player in metabolism remodeling both in solid and hematological malignancies. Interestingly, literature suggests that that a significant perturbation of metabolic pathways can be synthetically lethal with chemotherapy administration, thus managing to eradicate cancer where chemotherapy alone cannot. Two independent works on AML showed that therapy-resistant cancer cells display a similar metabolic response to two different drug settings. In details, in vivo cytarabine-resistant AML cells increase the levels of ROS and shifted their metabolism towards OXPHOS and lipid oxidation [243]. On the other hand, in vitro sorafenib-resistant AML cell lines showed a similar increase in ROS, with cells exhibiting a higher glucose demand, accompanied by a lower reliance on pentose phosphate pathways [244]. Interestingly, a switch towards mitochondrial metabolism was also reported for different solid tumors, namely colorectal, lung, prostate and breast cancer. In particular, in vitro models and patients-derived samples of colorectal cancer cells resistant to 5-fluorouracil were shown to shift their metabolism towards OXPHOS. This metabolic reprogramming is accompanied by an increase in ROS production and by a diminished lactate production and pentose phosphate pathway activity [245]. Similarly, in vitro and in vivo models of lung cancer were shown to increase mitochondrial mass and activity upon short-time cisplatin exposure: this metabolic shift is associated to a parallel downregulation of glycolytic pathway [246]. Analogously, docetaxel-resistant prostate cancer was shown to shunt several metabolic intermediates, namely lactate, glutamine, and glucose towards OXPHOS, with a concomitant decrease in intracellular ROS content [247]. Furthermore, doxorubicin resistance in a primary triple-negative breast cancer cell line was shown to be achieved through the OXPHOS upregulation, with a parallel reduction in lactate production [248]. Ultimately, Herlyn and collegues reported that upon cisplatin or vemurafenib treatment, the appereance of multidrug resistant melanoma cells rely on JARID1B up-regulation. The JARID1B high expression in turns induces sustained up-regulation of proteins involved in the electron transport chain and down-regulation of glycolytic enzymes [249].

In all the aforementioned models, the authors showed that OXPHOS inhibition can overcome the cancer resistance to chemotherapy.

Interestingly, the metabolic reprogramming towards mitochondrial activity was shown to be highly model-specific, with other solid tumors reacting in a completely opposite manner to chemotherapy. For example, gastric cancer was shown to upregulate glycolysis upon cisplatin treatment: in this setting, glycolysis inhibition through enolase downregulation or glucose starvation was shown to revert cisplatin resistance [250]. In addition, even if highly reliant on mitochondrial metabolism [251], doxorubicin-resistant anaplastic thyroid cancer was shown to activate the pentose phosphate pathway via the overexpression of 6-phosphogluconate dehydrogenase. Of note, the authors demonstrated that the inhibition of 6-phosphogluconate dehydrogenase can overcome the resistance to chemotherapy [252].

Along with carbohydrate metabolism, chemotherapy was reported to critically impact lipid-related pathways. In particular, two separate works on breast cancer revealed a significant increase in lipid droplets accumulation and cholesterol biosynthesis upon either doxorubicin [248] or tamoxifen [253]. It is worth noting that the silencing of perilipin, the proteic structural component of lipid droplets, resulted in reduced viability of doxorubicin-resistant breast cancer cells [248]. Lipid biosynthesis was also proven to be critical in other models of chemotherapy resistant cells. In particular, a recent work by Schreiber and colleagues showed in a panel of human tumors that the synthesis of polyunsaturated fatty acids is crucial for cancer cells to survive upon chemotherapy. Indeed, these lipids are substrates for the phospholipid glutathione peroxidase, whose activity prevents ferroptotic cell death [254]. On the other hand, fatty acid oxidation was shown to be a key player in ovarian cancer chemoresistance. Indeed, fatty acid oxidation both provides metabolic intermediates to fuel metabolism and sustains NADPH production: also here, the full reversion of the resistance to chemotherapy was obtained through fatty acid oxidation inhibition [255].

Eventually, glutamine metabolism perturbation was reported to be synthetic lethal with chemotherapeutic administration in several tumors. In particular, glutamine depletion was proven to be extremely effective on cisplatin-resistant lung cancer, eliciting massive death of tumoral cells that were not eradicated by the drug [256]. On the other hand, GBM cells were shown to survive upon rapamycin exposure through an upregulation of glutaminase activity, which allows cells to bypass the metabolic block imposed by rapamycin [257]. Suppression of either glutaminase expression or activity sensitized therapy-resistant GBM cells to rapamycin. Similarly, two independent works on ovarian and esophageal squamous-cell cancer revealed that glutaminase inhibition increased tumor sensitivity both to cisplatin [258,259] and paclitaxel [258]. Eventually, ovarian cancer cisplatin-resistant cells were shown to rely on MYC-dependent glutaminase upregulation to catabolize glutamine and fuel OXPHOS. Interestingly, the inhibition of glutaminase synergizes with cisplatin treatment, ultimately leading to increased ovarian cancer cell apoptosis [260].

As a proof of concept of the role of metabolism in regulating the response to a broad range of therapy agents, many works pointed out the extraordinary efficacy of coupling caloric restriction (i.e., a reduced calorie intake [261]) in empowering the response to standard of care therapeutic regiments. In particular, caloric restriction was demonstrated to be extremely effective in enhancing chemotherapy effects in breast cancer [262,263,264], lung cancer [265], colorectal cancer [266], glioma [267], and fibrosarcoma [262].

**Figure 3:**
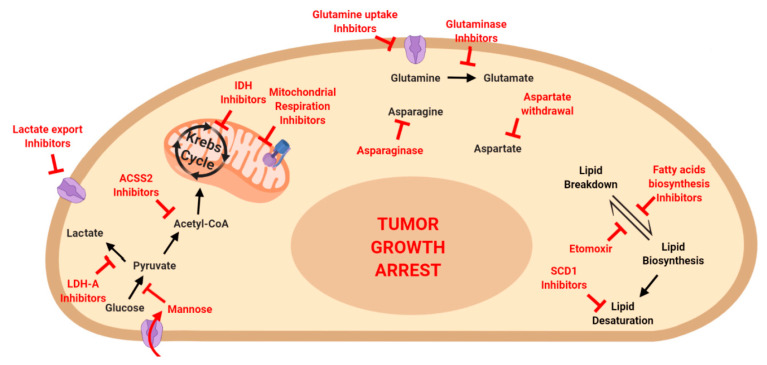
Targeting metabolic pathways to eradicate tumor growth. Inhibitors for several metabolic pathways have been described. The inhibition of glucose, amino acid and fatty acid metabolism was shown to be promising for a successful eradication of tumoral disease.

Finally, the metabolic addiction of cancer cells has been proposed as a therapeutic target per se (Figure 3), with metabolic drugs eliciting a profound impairment on tumor progression. Exploiting tumor-specific metabolic vulnerabilities might indeed represent a therapeutic strategy with minimal specific side-effects. One of the major mutations that induce metabolic reprogramming involves the enzymes IDH1 and IDH2. Since their identification, IDH inhibitors were proposed as efficient drugs to eradicate IDH-driven gliomas [268]. The metabolic addiction to IDH was also exploited in further works in AML setting, with the development of efficient drugs capable to counter tumor progression [269,270]. Moreover, the metabolic reprogramming generated by IDH mutation makes cancer cells extremely vulnerable to NAD depletion: indeed, a recent study by Cahill and colleagues showed that NAD deprivation leads to cancer regression in IDH-mutated GBM [271]. Besides IDH, other enzymes involved in glucose metabolism were shown to be exploitable to arrest tumor progression. Several works pointed out the efficacy of OXPHOS inhibitors in eradicating bot solid and hematological malignancies. In particular, the inhibition of mitochondrial ATPase was proven to counter GBM in vivo, without affecting neither fibroblasts nor astrocytes [272]. At the same time, the inhibition of electron transport chain complex I [273] and II [274] was show to suppress AML. In the same way, the impairment of mitochondrial respiration was reported to impede ovarian cancer progression [275], while the contemporary inhibition of glycolysis and OXPHOS was demonstrated to suppress breast cancer [276]. Similarly, the simultaneous abrogation of mitochondrial respiration and lactate export promotes cancer death in several in vitro cancer models [277]. Interestingly, the inhibition of lactate generation was as also investigated as a possible therapeutic strategy, however results were highly model-dependent. On one hand, inhibition of LDH-A was shown to critically reduce tumor burden in TP53-mutated pancreatic cancer, while TP53-wild type tumors were not affected [30]. On the other hand, inhibition of LDH-A enzyme was shown to be insufficient to eradicate melanoma, which activate the autophagy pathway in order to survive LDH-A inhibition [278]. Eventually, cancer addiction to glucose metabolism was recently exploited to treat tumor cells with the monosaccharide mannose. Mannose is uptaken via the same transporter as glucose, however its accumulation within cancer cells impair all the branches of glucose metabolism, thus leading to tumor shrinkage [279].

Besides glucose, amino acid metabolism was an intriguing field of investigation to identify metabolic vulnerabilities of cancer cells. The enzyme drug L-Asparaginase represents a cornerstone in acute lymphoblastic leukemia (ALL) treatment since it exploits cancer dependency on asparagine to counteract ALL progression [280,281]. However, a recent study suggested a repurposing of L-Asparaginase in breast cancer treatment, as asparagine bioavailability was shown to be crucial for metastasis spreading [140]. Besides asparagine, aspartate was also shown as a limiting metabolite in tumor growth, with tumors overexpressing asparaginase (which converts asparagine to aspartate) growing at faster rate [282]. The heavy addiction of cancer cells to amino acids was further shown by Jordan and colleagues, who proved that chemo-naïve AML stem cells undergo cell death when amino acid uptake and metabolism are pharmacologically impaired [283]. Similarly, the pharmacological inhibition of glutamine uptake was shown to be crucial for breast, lung, and colorectal cancer progression, with inhibited cells undergoing increased oxidative stress and tumor proliferation arrest [284].

Finally, fatty acid metabolism impairment represents an exploitable target in cancer therapy. Several studies pointed out that the fatty acid oxidation inhibitor etomoxir can counter tumor progression, both in solid and hematologic malignancies. In particular, etomoxir was proven to be efficient in blocking tumor growth and metastasis spreading in bladder cancer, with inhibited cells undergoing lipid accumulation and NADPH deprivation [285]. Moreover, etomoxir was reported to eradicate Myc-driven triple negative patient-derived breast cancer [286]. Similar results were obtained in GBM, with etomoxir leading to ROS accumulation and ATP and NADPH shortage, which in turn promote cancer cell death [287]. Eventually, etomoxir cytotoxic effects were also confirmed in in vitro models of AML, where the drug elicited a dose-dependent apoptosis induction [288]. Besides fatty acid oxidation, fatty acid synthesis was shown as a promising target in arresting tumor progression. Indeed, the knock-out of SREBP transcription factors, which regulate lipid biosynthesis, abrogates bladder cancer proliferation and migration phenotype [22,289]. In addition, a recent work focused on the role of acetyl-CoA in cancer progression. Tumor cells undergoing Warburg effect need to produce adequate amounts of acetyl-CoA, that is mainly shunted to lipid synthesis and histone modification: indeed, the absence of acetyl-CoA synthetase (ACSS2) enzyme leads to tumor burden reduction in in vivo models of hepatocellular carcinoma [290]. Similarly, two independent works demonstrated that the pharmacological inhibition of acetyl-CoA carboxylase enzyme leads to tumor growth arrest in lung [291] and pancreatic cancer [292]. Finally, fatty acid desaturation was also shown to be a critical step in cancer homeostasis, with the inhibition of the enzyme Stearoyl-CoA desaturase 1 (SCD1) leading to tumor growth impairment in colorectal [293] and lung and gastric cancer [294].

## 8. Conclusions

A completely abnormal metabolism has been historically indicated as one of the major hallmarks of cancer [96]. In this review, we have shown that cancer metabolic reprogramming plunges its roots into a genetically altered background, with mutations in major cell physiology regulators profoundly affecting the metabolism of tumor cells. In this scenario, the metabolic fluxes are heavily changed, with a major involvement of glycolysis and glutamine metabolism to fuel tumor growth, together with a dynamic lipid turnover. However, similar to what is observed under the genetic and transcriptional point of view, tumors revealed to be heterogeneous also in their metabolism, with cells in the same lesion adopting completely different pathways to thrive in the presence of different stimuli. Moreover, cancer cell metabolism is highly dynamic and continuously reprogrammed during tumor progression, from the spreading of metastatic cells to the colonization of new organs, to the formation of overt metastases. All of these steps require a precise fine-tuning of cellular metabolism, in a way that is dictated by both endogenous and exogenous factors. Eventually, cancer cells are capable of reprogramming their metabolic profile to survive chemotherapy, with resistant cells emerging upon the treatment (also) thanks to a bioenergetic shift. Fortunately, this profound addiction of cancer cells to metabolic pathways has provided quite a number of druggable targets that can be exploited in therapeutic settings in an attempt to hopefully counter tumor progression.
